# Real-Time Monitoring System for a Utility-Scale Photovoltaic Power Plant

**DOI:** 10.3390/s16060770

**Published:** 2016-05-26

**Authors:** Isabel M. Moreno-Garcia, Emilio J. Palacios-Garcia, Victor Pallares-Lopez, Isabel Santiago, Miguel J. Gonzalez-Redondo, Marta Varo-Martinez, Rafael J. Real-Calvo

**Affiliations:** 1 Electronic Technology Area, University of Cordoba, Cordoba 14071, Spain; p92pagae@uco.es (E.J.P.-G.); vpallares@uco.es (V.P.-L.); el1sachi@uco.es (I.S.); el1gorem@uco.es (M.J.G.-R.); rafael.real@uco.es (R.J.R.-C.); 2 Department of Applied Physics, University of Cordoba, Cordoba 14071, Spain; fa2vamam@uco.es

**Keywords:** Photovoltaic distributed generation, power quality, real-time monitoring, embedded systems, wireless sensor network (WSN)

## Abstract

There is, at present, considerable interest in the storage and dispatchability of photovoltaic (PV) energy, together with the need to manage power flows in real-time. This paper presents a new system, *PV-on time*, which has been developed to supervise the operating mode of a Grid-Connected Utility-Scale PV Power Plant in order to ensure the reliability and continuity of its supply. This system presents an architecture of acquisition devices, including wireless sensors distributed around the plant, which measure the required information. It is also equipped with a high-precision protocol for synchronizing all data acquisition equipment, something that is necessary for correctly establishing relationships among events in the plant. Moreover, a system for monitoring and supervising all of the distributed devices, as well as for the real-time treatment of all the registered information, is presented. Performances were analyzed in a 400 kW transformation center belonging to a 6.1 MW Utility-Scale PV Power Plant. In addition to monitoring the performance of all of the PV plant’s components and detecting any failures or deviations in production, this system enables users to control the power quality of the signal injected and the influence of the installation on the distribution grid.

## 1. Introduction

Within the framework of renewable energies, photovoltaic (PV) is one of the technologies with the greatest future projection. Its numerous advantages, such as simple installation, high reliability, zero fuel costs, very low maintenance costs, and the lack of noise due to the absence of moving parts [1], have resulted in a high growth rate. Indeed, nowadays PV represents the third-largest source of renewable energy after hydro and wind [2]. Since 2010, the world has added more solar photovoltaic capacity than in the previous four decades. Market researchers [3,4] forecast another year of solar growth for 2016, when new installations are expected to reach 69 GW—up from 59 GW for 2015. A cumulative global PV installed power is expected to surpass 310 GW by the end of 2016 [4]. Opportunities for improvement in this sector are not based solely on increasing the level of installed capacity, but also on the necessity of embracing the challenges of technological advancements that allow producers to both optimize production in order to reduce generation costs, thus rendering PV energy more competitive, and also enable them to make this type of electricity production dispatchable, so that it can be integrated into the future context of Smart Grids [5,6].

### 1.1. Brief Overview and Key Innovations

Optimizing the production process and the reliability of photovoltaic systems requires plants to increase production, as well as the lifetime and availability of all of their elements, while reducing operating and maintenance costs (O&M) [7,8,9,10]. Since solar installed capacity is growing continuously, a small percentage improvement would imply significant net progress and, in general terms, a reduction in these facilities’ costs. Such systems cannot work efficiently if operations are not automated [11]. For this purpose, a new system, named *PV-on time*, developed and installed in a Grid-Connected Utility-Scale PV Power Plant, is presented in this paper. The system developed here provides a detailed comprehensive real-time supervision of the performance of all the components, quantifying different loss mechanisms and detecting the presence of any faults or deviations by comparing the production of similar elements in the plant.

On the other hand, such facilities must overcome the disadvantages associated with the stochastic nature of this type of energy production, linked as it is to the presence of uncontrollable weather events. Such events originate ramp ups/downs in solar plant power output [12] that are difficult to predict. This is one of the main disadvantages of this type of renewable facility, especially in terms of participating in the energy market. Nowadays, we must face the challenge of a dispatchable photovoltaic energy, improving the optimal balance between generation and demand at all times, thus ensuring the stability of the grid. This problem could be overcome, as it has been planned here, with the use of storage systems wherein the real-time management of the power flows involved in the electrical system is needed to ensure coverage of demand. The use of storage systems, one of the most widely accepted solutions to ensure the coverage of electricity demand [13], requires a detailed knowledge of the plant production mode in order to optimize the investment of economic resources in such systems—a problem not yet properly resolved, but which will be an inevitable part of PV facilities in the future [12,14,15,16]. Moreover, the implemented system not only enables a distributed record of the operating parameters of the various devices comprising the installation, as well as a record of the energy signals produced at different points in the system, but also the monitoring of a large set of the environmental magnitudes. Thus, with this solution, a weather forecasting is possible with the aim to correlate the relationship between the plant performance, real-time production, and the environmental parameters.

Furthermore, in the present context, consumer requirements have increased the demands on the quality and reliability required of the power generated [17], mainly in the framework of future large-scale integration in the grid of distributed renewable energy [18,19]. Therefore, in this project, not only is it necessary to increase and secure production, but also to improve the quality of the signal produced and injected into the grid by such facilities. This must be combined with the determination of the named hosting capacity of low and medium voltage grids for the production of PV installations [20]. The grid capacity can also be improved by means of the storage systems, among other measures. This may prevent the grid from having to be resized in order to withstand the total installed distributed energy. As indicated in [21], the significant growth of grid-connected PV systems has not been accompanied by the implementation of techniques for diagnosing, monitoring, controlling, and detecting faults in these facilities. This highlights the challenge of this work—despite the importance of having an effective failure control, the majority of PV plants in operation have no supervision mechanisms available. Diverse studies have addressed the development of applications for analyzing the operation of Utility-Scale PV Power Plants, as indicated in [22,23], as well as diagnosing the performance of PV solar modules [24,25]. Unlike this project, the cross-sectional studies provide little information relevant to the power quality (PQ) of the signal injected into the grid, and the majority have a log interval limited to monitoring frequency of the inverter.

The *PV-on time* system improves upon the existing literature in several ways. The main feature that differentiates this system is that, with the additional sensors distributed in the plant, it is possible to obtain a more complete diagnosis of the energy signal injected to the grid than those provided by inverters, including PQ analysis in real-time (on time).The principal objective of this system is to increase the sampling frequency in the measures to accomplish the PQ standard, given that inverters are not able to reach these time requirements.

Another novel characteristic of the developed system is that the data acquisition equipment has been integrated with a protocol for precisely synchronizing the data collection, establishing an accurate time correlation among events in the plant. For this aim as a method of synchronism, the IEEE 1588 standard Precision Time Protocol (PTP) is used [26]. PTP synchronism properly establishes a temporal framework for the comparison between the measured parameters in various inverters. Moreover, when the protocol is joined with the interconnection to a Gigabit Ethernet Switch with BoundaryClock, it delivers the White Rabbit protocol, which enables nanosecond precision and distances up to 10 km. Thus, this synchronization technology means a disruptive impact on the photovoltaic sector, due to the accurate synchronization and the high degree of determinism reached. This novel technology is being considered in critical infrastructures [27,28].

In addition, a complete and detailed supervision of the behavior and operating mode (planned here for this PV facility) requires a system for automating the processing, management, and analysis of all the information. Developing and refining this infrastructure was also the main objective of this work. To this end, an application for on-line monitoring of the performance of all of the PV plant’s components, the deviations in production, the PQ of the signal injected, and the influence of the installation on the distribution grid has been incorporated in the *PV-on time* system.

So, taken as a whole, this development is an important step towards a real dispatchability of PV facilities and a distributed energy generation. It may be the case of large PV installations, which often have several hectares of extension [29]—usually a PV power plant requires between one and two hectares per each 1 MWp deployed [30]. The effects at a large-scale facility can affect the PQ and the reliability [31,32,33,34,35]. Therefore, it is a great advantage to analyze the production in real-time and with both a high temporal resolution and a high degree of positive correlation over the total surface. Another field of application, where this intelligent infrastructure might be justified, is the co-allocation of several different solar power stations developed on adjacent sites. In this situation, an accurate synchronization of the PV plants is necessary for a correct interplay between power sources, despite their geographical separation [36]. It is a fundamental issue for the distribution system operator (DSO) to be fully aware of the production of each feeder for adequate decision-making. Moreover, the system also aims to be used as a research tool for validating power loss models in PV panels due to temperature—establishing the relation between PQ events and plant events, determining wiring losses in face of the wireless sensors, dispatchability analysis, reducing O&M costs, and improving the monitoring system itself.

### 1.2. Outline of the Paper

The rest of this paper is organized as follows. A full description of the developed system is presented in Section 2: in Section 2.1, the embedded control and acquisition system installed in the inverters to record and process DC and AC voltages and currents measurements in real-time is presented; in Section 2.2, a wireless sensor network (WSN) added in order to register environmental magnitudes and energy production is addressed; Section 2.3 describes the weather station installed in order to register environmental measures; Section 2.4 explains the synchronization technique of all the measurements within the PV plant; Section 2.5 briefly describes the communication system used in *PV-on time* and introduces another communication system based on the IEC 61850 standard, which will be integrated in future work. Section 2.6 presents a full overview of the developed monitoring and processing system to supervise the plant’s operation in real-time. Section 3 studies the performance of the *PV-on time* system in a transformer center (TC) belonging to the Utility-Scale PV Power Plant under examination. Finally, the conclusions are given in Section 4.

## 2. *PV-on Time* System Description

**Figure 1:**
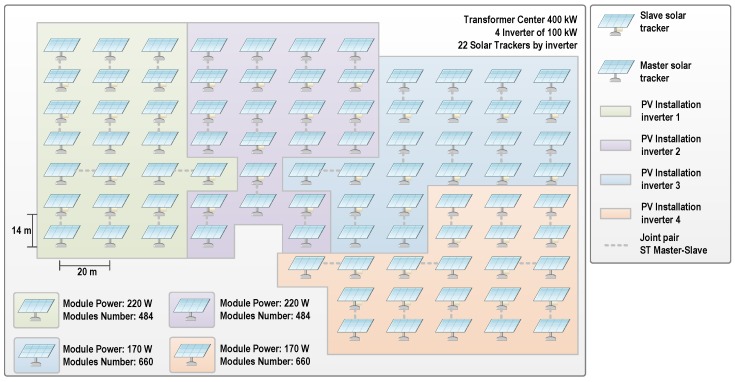
Monitored photovoltaic (PV) modules distribution. ST: Solar Tracker.

The scope of the *PV-on time* system was defined in terms of the topology of the Grid-Connected Utility-Scale PV Power Plant in which it has been installed, tested, and tuned. It is an installation with a nominal power of 6.1 MW located in Cordoba, Spain, and a property of the *Magtel Operaciones SL* company. Specifically, the first prototype system has been developed to monitor a TC which provides a nominal power of 400 kW. The TC consists of four inverters (INV), each one connected to 22 solar trackers (ST). The PV modules supervised are of different manufacturers: those belonging to INV1 and INV2 have an individual nominal power peak of 220 Wp and there are 484 PV modules connected to each inverter; whereas those belonging to INV3 and INV4 have an individual nominal power of 170 Wp with 660 PV modules connected to each inverter. The total number of PV modules being monitored and the STs’ distribution associated with each inverter are shown in Figure 1. STs have been grouped into pairs, one working as a master and the other as a slave. 

**Figure 2:**
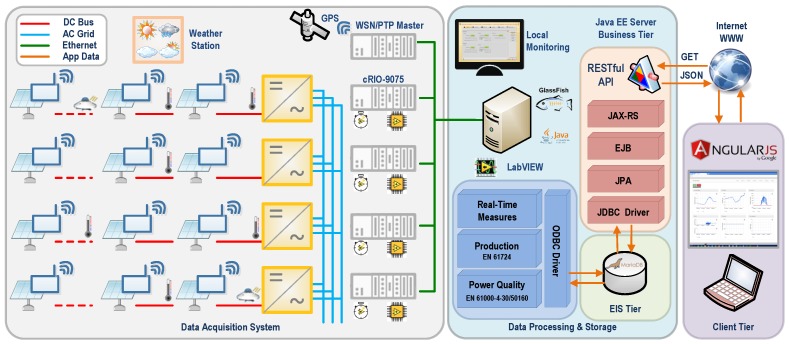
Schematic structure of the real-time monitoring system. Devices, conceptual blocks, communications, and information flow. EIS: Enterprise Information System; EJB: Enterprise Java Beans; JAX-RS: Java API for RESTful Web Services; JDBC: Java Database Connector; JPA: Java Persistence API; JSON: JavaScript Object Notation; ODBC: Open Database Connectivity; PTP: Precision Time Protocol; RESTful: Representational State Transfer; WSN: Wireless Sensor Network.

In this section, the above-mentioned methods and parameters selected for the implementation of the monitoring system *PV-on time* for the solar system are detailed. The structure of the developed system comprises sensors, programmable controllers, and a monitoring and supervision system, along with the different relationships between them (Figure 2).

In *PV-on time* system, the data acquisition was performed using the real-time programmable controller CompactRIO 9075 (cRIO, National Instruments, Austin, TX, USA). The cRIO, manufactured by National Instruments (NI), has already been used successfully in prior works [37,38,39]. The data were transferred to the cRIO by both wired and wireless communications. In the developed system, four cRIOs were connected to the inverters to measure electricity production and PQ magnitudes [40,41]. Another cRIO was used for monitoring environmental parameters and electrical parameters in PV field using a wireless mechanism. The data timestamp correlation was established by means of PTP synchronism. For this goal, a global positioning system (GPS) module was integrated into the cRIO that performs wireless measures and was configured as a master in the synchronization process. Other cRIOs, attached to the inverters, were connected to the local ethernet network and were configured as slaves. In this way, the data acquisition system can keep a unique distributed timestamp with a high precision. Finally, the integration and control of all measurement devices was carried out by the monitoring system, calculating performance magnitudes [23] and PQ parameters in different temporal scales, as detailed below. This section gives details on the different system components and their implementation in the experimental setup categorizing them into six subsections; *i.e.*, wired monitoring in inverters, wireless monitoring in a PV field, weather station for monitoring environmental magnitudes, and the synchronization, communication, and monitoring systems, which are systems needed for the correct processing of all measurements.

### 2.1. Measurements in Inverters: Smart Power Quality Analysis

**Figure 3:**
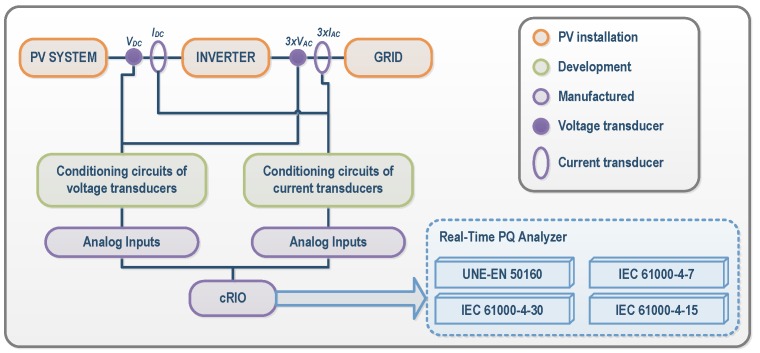
General structure for electrical measurements in inverters. cRIO: CompactRIO programmable controller; PQ: Power Quality.

The general structure of the developed acquisition system installed inside an inverter is shown in Figure 3. By incorporating additional sensors, measurements of continuous (DC) currents and voltages were performed at the input of each of the four inverters of the TC selected in the Utility-Scale PV Power Plant. Similarly, the alternating currents (AC) and voltages were also measured in the three-phase output of the four inverters. Conditioning circuits for the voltage and current transducers used for performing DC and AC measurements in the inverter were previously designed and manufactured in our University laboratory and are described below.

**Figure 4:**
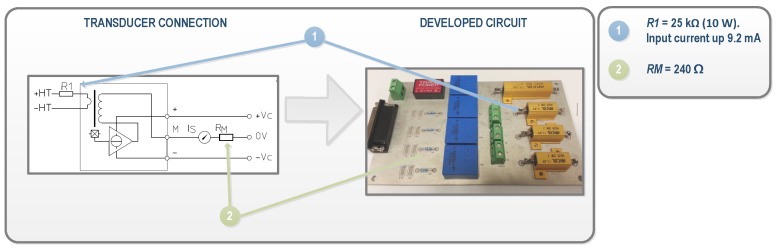
Signal Conditioning of the DC and AC voltage transducers. RM: multi-turn precision resistor.

For DC and AC voltages, the LEM LV 25-p transducer (LEM, Fribourg, Switzerland) was selected. It is a closed loop Hall effect current transducer. The conditioning circuit developed for voltage measurements using this transducer is shown in Figure 4. In the circuit, a current proportional to the measured AC voltage must be passed through an external resistor R1. According to the transducer datasheet, a voltage of up to 500 V can be measured, and a 25 kΩ was selected as R1 to transform the AC voltage in a nominal current of the 10 mA at full scale. At the output block, high precision resistors of 240 Ω were selected in order to transform the current into a voltage within the range of the acquisition card (RM in Figure 4). In the case of DC voltage measures at the input of the inverters, the LEM LV 25-P is also used and can measure up to 700 VDC. So, the conditioning circuit was the same as used in AC measures, but allowing the maximum voltage. Consequently, a 50 kΩ resistance was selected as R1 to reach 12 mA DC current. This current is proportional to a 25 mA output current. This configuration is suitable to measure the maximum DC voltage from PV-strings, which is 600 V.

**Figure 5:**
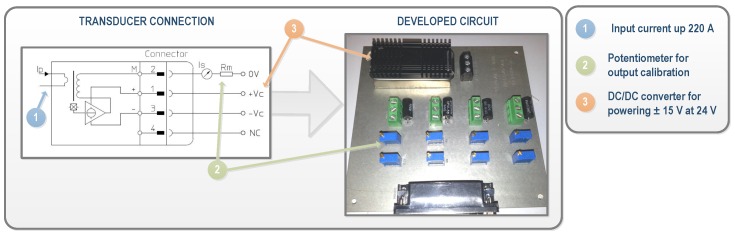
Signal Conditioning of the continuous (DC) and alternating (AC) current transducers.

Regarding currents, closed loop Hall effect current transducers were also used. The LA 305-S transducer and LF 305-S (LEM, Fribourg, Switzerland) were selected for DC and AC currents, respectively. The LA 305-S ensures that the maximum DC current generated in the plant, which is about 220 A, can be measured, while it has a diameter suitable for the section of the plant’s conductors. The operation range of the LF 305-S is up to 300 A, which is higher than the inverters’ output, whose maximum line nominal currents are 140 A. A conditioning of current measurements was needed to adjust the levels for the data acquisition target. Figure 5 shows an example of the conditioning circuit of the developed current transducer. In this case, the output range is selected and calibrated using multi-turn precision resistors, labeled as RM in the figure.

**Figure 6:**
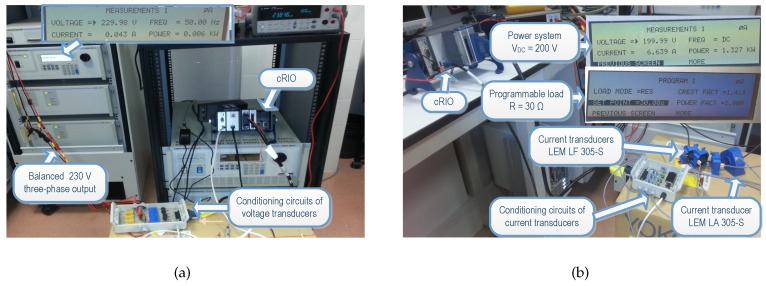
Calibration of the conditioning circuits developed joint to the CompactRIO programmable controller (cRIO): (**a**) Test for voltage transducers; (**b**) Test for current transducers.

Considering design conditions in the steady state, the conditioning circuits systems for measurements of voltage, current, and three-phase power were calibrated. The completed measurement system, conditioning circuits joint to a cRIO, were calibrated for the verification of powers algorithms processing. The equipment used for these calibration tests was composed of an AC programmable power system, a programmable active load, and a cRIO with an analog inputs module connected to the output of the power system. The AC programmable power system, AC and DC model 9003iX of California Instruments (AMETEK Programmable Power, San Diego, CA, USA), integrates a transient network analyzer and a three-phase output; it can also run several standardized PQ tests (voltage sags, swells, flickers, *etc.*). Joint to the active load (3091LD model of California Instruments) it was able to generate different signal levels in order to reproduce the behavior of the inverter output. So, each conditioning circuit was calibrated as if it was located in the photovoltaic plant. Using different power generation levels, it was possible to determine the quality of the measurement system over a wide dynamic range, and also to verify the stability for different conditions of signal generation. A program was designed for this purpose and executed locally on the cRIO system. Figure 6 shows a calibration test of the measurement system.

Once the conditioning circuits were carefully calibrated in the laboratory, their correct operation in the PV plant was also verified. The cRIO integrates the acquisition modules connected to the sensors previously mentioned, which are necessary for measuring electrical signals in the input and output of each inverter. The acquisition modules are the NI 9215 models and include four simultaneously-sampled analog input channels. They have a sampling rate of 100 kS/s with NIST-traceable calibration and a channel-to-earth ground double isolation barrier for security and noise. They guarantee the computational accuracy needed for both measuring PQ parameters and for detecting critical PQ events in real-time. With this resource, the system can act as a PQ meter in the inverter output, as well as generating information on production in the input and output of inverters.

DC and AC voltages and currents measurements were acquired according to UNE-EN standard 61724:1998 [42] for monitoring energy production in PV systems [43,44,45,46]. Furthermore, they were registered according to IEC standard 61000-4-30 [47] for PQ analysis and to synchronization standards [26], one of the original features incorporated in the *PV-on time* system.

**Figure 7:**
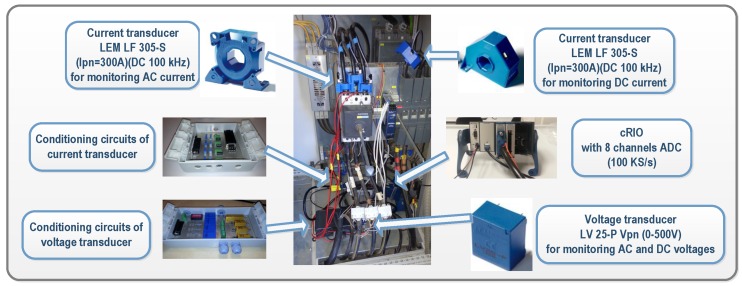
Electrical measurements performed in inverters in grid-connected PV plant.

In cRIO models, a deterministic real-time processing of all information and data was possible by means of the real-time operating system (RTOS) integrated into their chassis. In addition to PQ events and steady state signal variations such as harmonics and voltage unbalance, power and energy production were also determined in the RTOS from voltage and current measurements performed according to several electromagnetic compatibility standards [46,47,48,49]. Figure 7 shows the developed acquisition system installed in the PV plant.

### 2.2. Wireless Sensor Network

In the Utility-Scale PV Power Plant, a WSN was also added in order to register environmental magnitudes and the energy production related to INV2. For this, autonomous devices and independent nodes equipped with sensors to monitor physical or environmental magnitudes were installed. These sensors give greater flexibility, incur lower costs, and provide the ability to create intelligent WSN systems. WSN technology allows the analysis of the difference (in an O&M framework) between wired and wireless infrastructures [22].

**Figure 8:**
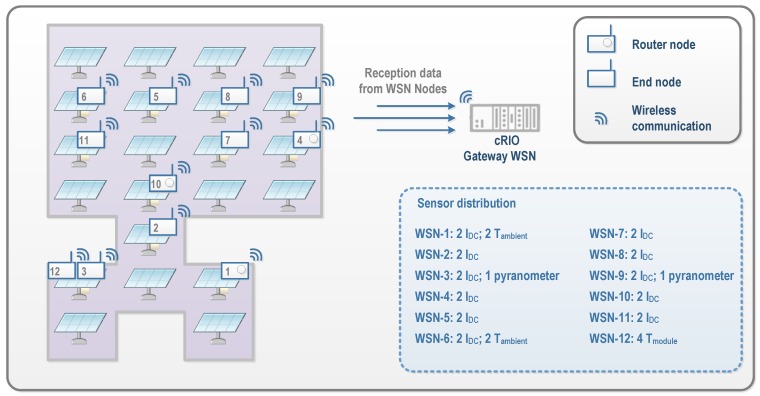
Relationship between the sensors and the wireless sensor network (WSN) nodes.

The *PV-on time* system uses the National Instruments WSN (NI-WSN) platform, consisting of three main components: nodes, gateways, and programmable controller cRIO. The NI-WSN3226 nodes used for the measurements have direct connectivity to four sensors. The wireless network scale can range from tens to hundreds of nodes and it integrates easily with existing wired measurement and control systems. In the NI-WSN system, an NI-WSN9795 gateway acts as the network coordinator, responsible for configuring all distributed nodes and collecting measured data from all of them. The gateway is a module installed in another cRIO, which was also configured to work as the PTP master. NI-WSN parent devices (routers and gateways) can only have a maximum of eight end nodes connected to them at a given time in the star topology. This limitation could be a problem when a device configured as an end node might not be able to join a router node. To avoid this, a reliable and efficient WSN meshing configuration that can connect up to 36 measurement nodes to a single WSN gateway was installed. For this, a distance test was undertaken in order to evaluate the possible interferences of several components (ST, TC where gateway was installed, inverters, and metallic structures) when each WSN node was installed. No significant interferences were detected, with all messages delivered to the gateway. This mesh topology was deployed according to the structure of the Utility-Scale PV Power Plant, in which STs are distributed in parallel and the distance between them can be either 14 or 20 m. To ensure communication quality, three nodes were configured as mesh routers, as shown in Figure 8.

**Figure 9:**
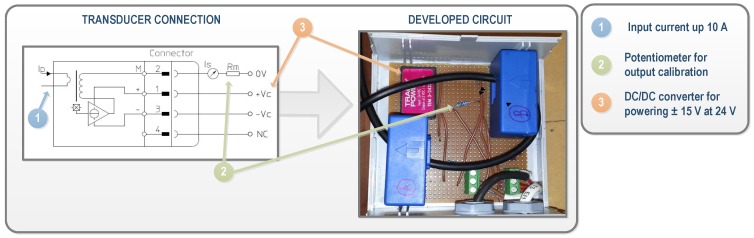
Conditioning of the current transducers for measuring the DC currents generated by the solar trackers.

**Figure 10:**
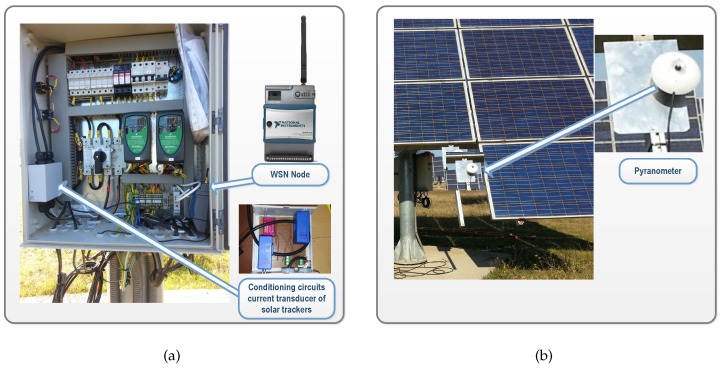
Photovoltaic measurements performed in solar trackers in grid-connected PV plant: (**a**) WSN node and DC current sensors; (**b**) Detail of a pyranometer.

As can be seen in Figure 8, 12 spatially-distributed wireless nodes installed on slave STs associated to INV2 were established as interface with the sensors. Two direct current sensors for measuring the currents generated by the master–slave pair were installed in each slave. These measures enable the wiring losses in strings to be calculated. The WSN nodes inputs connected to current sensors were configured as generic ±10 V voltage inputs. For this aim, the LEM LA 125-P sensor (LEM, Fribourg, Switzerland) was selected. This model ensures an adequate conditioning to the section of the conductors (Figure 9). The employed conditioning circuit has also been designed, developed, and calibrated in our University laboratory specifically for this application. Figure 10a shows a WSN node and the currents conditioning circuit, whose topology is equivalent to the one used for the inverters’ currents.

To determine rates of standard PV installations production and some of the mechanisms of losses in PV production, it is necessary to know the solar radiation collected by the PV modules as well as ambient temperatures and PV modules.

**Figure 11:**
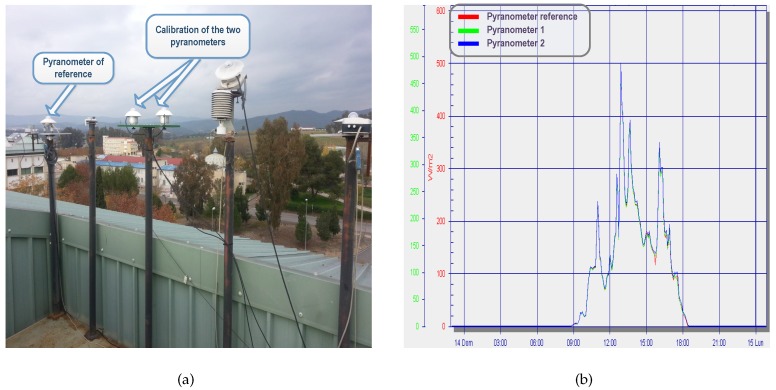
Calibration of the pyranometers: (**a**) Detail of the pyranometer on the radiometric installation; (**b**) Irradiance data recorded during a day.

For measurement of solar irradiance, two pyranometers GEO-SR12 [50] (Geonica, Madrid, Spain) were installed on opposite sides of the PV plant corresponding to the INV2. Both pyranometers were installed parallel in the plane to STs (see Figure 10b). For irradiance level measurements, a loop current (4 to 20 mA) with a resistance of 500 Ω was used. Pyranometers are powered by a 24 V power supply from the electrical panel. The WSN nodes inputs connected to current sensors were configured as generic ±10 V voltage inputs. Previously, the two pyranometers were installed on the roof of the Department of Applied Physics at the University of Cordoba and they were calibrated. Firstly in the calibration process, the relative difference between the irradiance measured by the two new sensors was calculated point by point. The average value of the relative difference between the two measures of the new sensors was 0.89%. This value is lower than the calibration uncertainty specified by the manufacturer, which is fixed at 1.8%. So, the calibration of the pyranometers was considered correct. Secondly, the relative difference between each of the two new sensors with other reference sensor GEO-SR12 was calculated (see Figure 11a). The average value of the relative difference between the measurements of the new sensor 1 and the new sensor 2 with the reference sensor of the radiometric station was 2.25% and 2.23%, respectively. Again, the relative differences were very small, so they were acceptable and within the error of the sensor itself. In Figure 11b, irradiance data recorded by the sensors during a day have been represented. The two new sensors of the *PV-on time* system are represented in blue and green and are compared with the reference sensor (in red). Despite the instability of this day, the calibration results depicted a high degree of accuracy in the measurements.

Regarding temperature, four PT100 sensors for measuring ambient temperature and four surface-mount PT100 registering the solar module’s temperatures were installed. This type of sensor guarantees an accuracy of about ±0.2 °C. For this, the inputs were configured as specialized temperature measurement inputs with a resistance temperature detector (RTD).

### 2.3. Weather Station

A WMRS200 Oregon Scientific weather station was installed in the Utility-Scale PV Power Plant, registering the rain index, wind speed and direction, atmospheric pressure, and ambient temperature. It makes use of wireless remote sensors and has a USB port in order to be connected to a computer, and works with software from the manufacturer to show the collected weather data. In this project, however, Cumulus software was used in order to visualize data. Cumulus has the advantage of being a freely-distributed software for data retrieval, storage, and display from an electronic weather station, and it is easy to access weather registers along with daily, monthly, and annual values, as well as maximum and minimum data. Such environmental magnitudes enable production performance indexes in the modules associated to the four inverters to be calculated [23].

### 2.4. Synchronization System

**Figure 12:**
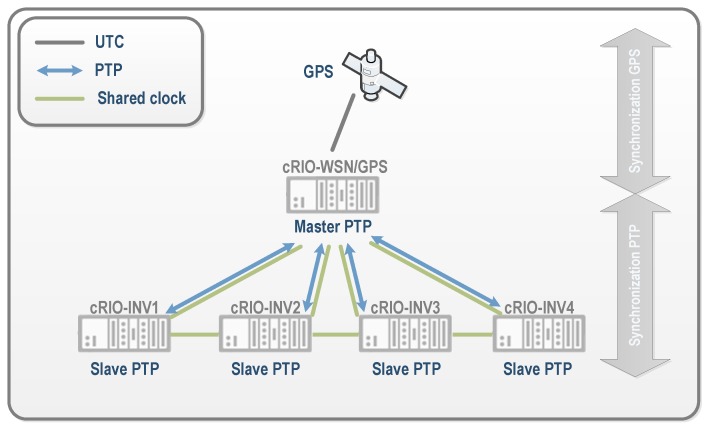
Synchronization system scheme. UTC: Universal Time Coordinate; PTP: Precision Time Protocol; GPS: Global Positioning System; cRIO: CompactRIO programmable controller; INV: Inverter; WSN: Wireless Sensor Network.

In the *PV-on time* system, a GPS module has been integrated into the cRIO chassis. This cRIO is responsible for collecting measurements of wireless nodes in order to maintain a universal time reference with high precision for all wireless measurements. The GPS module allows all the wireless measurements to be time stamped with a universal base. Furthermore, this same cRIO acts as the master in the PTP [26] to synchronize the clocks of the four cRIO systems installed in the substation, which are responsible for all actions of currents, voltages, and PQ measurements in the inverters (Figure 12). PTP standard was developed to synchronize any type of equipment in industrial areas [39], and it is an evolution of the network time protocol (NTP) with further stability guarantees. This process ensures synchronization errors of approximately ±50 *μ*s. This supposes an error of 0.005% in the time stamping of wireless measures that have a sampling rate of 1 s and an error of 0.025% for PQ measures that have a sampling rate of 200 ms. This ensures a proper correlation of all measurements in the photovoltaic plant.

Among the most critical tests implemented were those related to the operating synchronism of all of the equipment integrated into the *PV-on time* system. Comment on this fact was made in a previous works [38,39], and further consideration will be given in future publications, since a correct synchronism is crucial for correctly establishing relationships between events in the Utility-Scale PV Power Plant, mainly those related to PQ events. As was discussed above in Section 1, PTP synchronization means a disruptive improvement in the performance of such facilities, as occurs in other scientific and industrial sectors. A substantial number of works based on the White Rabbit protocol [27,28], combining Synchronous Ethernet and PTP, have been applied to calibration and time-stamping with satisfactory results.

### 2.5. Communication System

The communication system is based on the single-process shared variable to transfer data between the high-priority loop and the low-priority loop, reducing the latency greatly. In the *PV-on time* system, voltages and currents of the inverters are captured in a high-priority loop. Then these signals are transferred to the low-priority loop to perform the PQ analysis. The low-priority loop logs the PQ measurements and writes the updates to a network-published shared variable for the subscriber at the server.

However, this communication system does not operate in real-time. To improve the determinism in data transfer, the communication system is being migrated to the IEC 61850 standard. This is the standard for communication networks and systems for power utility automation. The use of this technology provides smart distributed energy, allowing the integration of intelligent devices to communicate in real-time [51,52] and do so with a high degree of stability, coordination, and synchronization [38,53].

The IEC 61850 standard is based on the definition of a hierarchical data model, modeling all elements present in the system, both the physical devices and the functions performed by them. The upper level of the hierarchical model occupies the physical device and represents the visible behavior of it. A physical device can hold one or more logical devices (LD), whose mission is to represent information on a series of specific application functions. These functions are modeled using the concept of the logical node (LN). Thus, an LD can hold one or more LN, according to the functions performed. LN group data (data objects, DO) concerning the role modeled for them. Each data object may contain one or more data attributes (DA) which will be one of the basic types predefined in section 7-2 of the standard [54], such as boolean, integer or float, to mention but a few.

**Figure 13:**
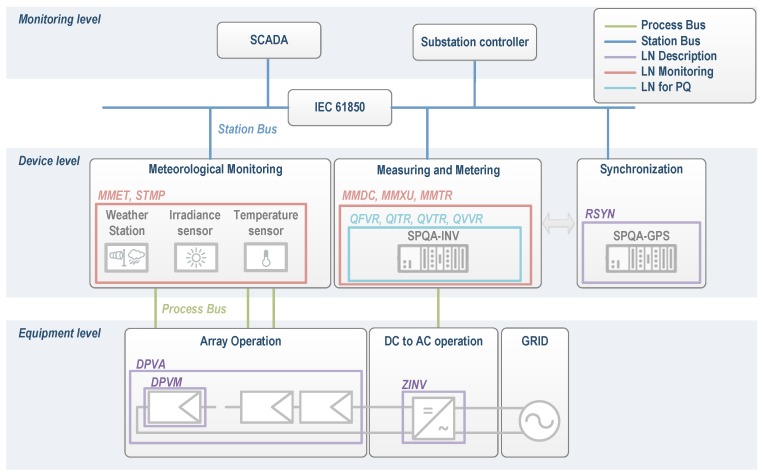
Overview of logical devices and logical nodes for distributed energy resource (DER) in the *PV-on time* system. DPVA: PV Array characteristics; DPVM: PV Module ratings; MMDC: DC Measurement; MMET: Meteorological Information; MMTR: Metering 3 Phase; MMXU: Measurement; QFVR: Frequency Variation; QITR: Current Transient; QVTR: Voltage Transient; QVVR: Voltage Variation; RSYN: Synchronism-check; SCADA: Supervisory Control And Data Acquisition; SPQA: Smart Power Quality Analyzer; ZINV: Inverter for converting direct current to alternating current (DC → AC).

Specifically, this paper presents the preliminary work focused on section 7.3 of IEC 61850-7-420 “Logical nodes system for photovoltaic (PV) logical device” [55]. This is devoted to LD and LN associated with the experimental PV plant under supervision. Figure 13 illustrates the defined LN for the *PV-on time* system and its relation to devices integrated into the same.

#### Communication Services IEC 61850

Communication services are a set of services and responses to them that allow the exchange of information within the system and enable different devices to behave identically from the point of view of network behavior. The services are defined using an object-oriented modeling technique. Specifically, the abstract communication services (ACSI) are defined by part 7-2 of the standard [54]. The concept “abstract” means that the definition of services focuses on what these services provide, not how they do it.

**Figure 14:**
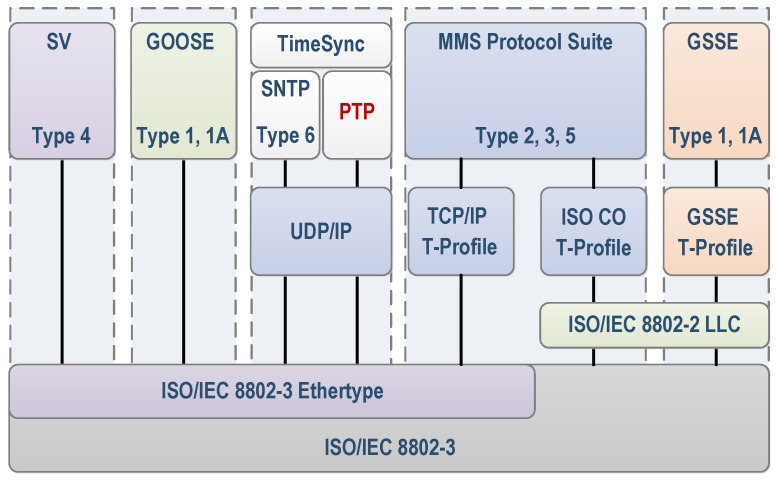
International Electrotechnical Commission (IEC) 61850 communication profiles [57]. GOOSE: Generic object-oriented substation event; MMS: Manufacturing Message Specification; SNTP: Simple Network Time Protocol SV: Sampled values.

Table 5 of the standard 7-2 [54] lists the ACSI models and their partners, such as generic substation events (GSEs) and transmission of sampled values (SV) services. The GSE model is very important because it supports the implementation of real-time applications. Generic object-oriented substation event (GOOSE) messages are associated with GSE services. GOOSE messages are based on a model talk around and are not compatible with the TCP/IP protocol. MMS messages are based on the TCP/IP standard and they are compatible with existing communication networks. The TimeSync messages use the UDP protocol that ensures greater transfer speed. This paper proposes the use of PTP protocol (IEEE 1588) for synchronization of the equipment by ensuring a synchronized transmission between transmitters and receivers of GOOSE messages. Figure 14 shows the different communication profiles defined in the standard.

**Figure 15:**
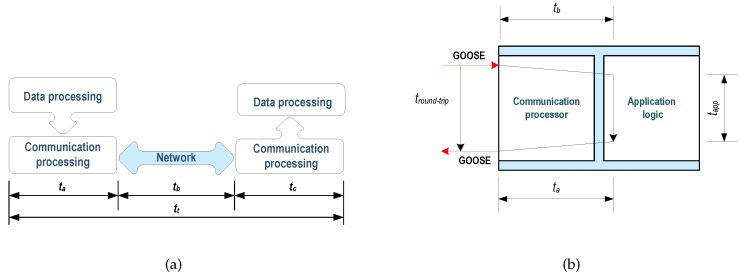
Methods for performance measurements according to IEC 61850: (**a**) Definition of overall transfer time [56]; (**b**) Round-trip time [60].

The definition of transfer time included in the IEC 61850 standard specifies that it comprises the complete transmission of a message (including processing time) on both sender and receiver sides. Accordingly, the transfer time counts from the time at which the sender makes the data available to the communications stack for transmission and ends at the instant at which the receiver extracts data from the communications stack. In Part 5 of the standard [56], the transfer time is divided into three parameters: $t_{a}$ and $t_{c}$ are the processing time on the sides of the transmitter and receiver, respectively, while $t_{b}$ is the time that the communications network needed to transmit data. Total time $t_{t}$ transfer is the sum of three times (Figure 15a). According to this part of the standard, the maximum time allowed for the exchange of information in the communication system is essential to establish performance requirements.

The maximum transfer time for the exchange of information is used in this part of the standard to classify different types of messages according to performance classes. Table 1 of Part 5 of the standard [56] lists seven classes of performance. The transfer time for protection and control applications classes with a transfer time lower than 10 ms, are defined for the most demanding applications in terms of response time. The time involved in the communication between devices within the substation has also been specified in other standards, such as IEEE Std 1646-2004 [58] and IEEE Std C37.115-2003 [59]. Both standards specify the supply time of the message as an indicator of communication performance. Similarly, IEEE Std 1646-2004 defines requirements for qualitative performance communications interfaces. Thus, for very high-speed messages, the delivery time should be less than 2 ms while for high-speed messages, it must be between 2 and 10 ms.

When measuring performance in a communications system IEC 61850, generally the end user does not have access to data and the internal resources of commercial devices. Looking ahead to measure the transfer time, as described in Part 5 of IEC 61850 [56], this is a difficulty. To resolve this, various tests have been proposed that allow measurement and/or estimation. Among these are the Round-Trip Test, Ping-Pong test, and Rally test [61].

The Round-Trip Time is a measure of the speed of a device [56]. Test equipment (test set, TS) and the device under test (DUT) are needed. In this case, the DUT receives a GOOSE message to which you subscribed, which it processes and publishes another message in response. The time spent since the GOOSE message subscribed until the other message is posted provides the Round-Trip time ($t_{round-trip}$). This time is the sum of three times (Figure 15b), $t_{round-trip}$ = $t_{a}$ + $t_{b}$ + $t_{app}$, $t_{b}$ being the time the device needs to receive the subscribed message. In other words, the time which the communication stack needs to process the received message. Similarly, $t_{a}$ is the time needed for the communications stack to process the outgoing message, and $t_{app}$ is the time related to the logic required to accept the incoming message and generate outgoing.

### 2.6. Monitoring and Processing System

Finally, a system was developed for remotely controlling the plant. The design of the data processing system was based on the cRIO data acquisition system and on the LabVIEW programming software, both from NI. The *PV-on time* system also possesses a web application for supervising all of the equipment and for automating the processing, management, and analysis of all recorded information. The architecture of the developed system was designed to be easily scalable and configurable. That is, it was structured so that it can be easily adapted to different Utility-Scale PV Power Plant topologies and to different data measurement equipment.

#### 2.6.1. Data Processing

The LabVIEW system is able to process the information in two levels. Firstly, the most priority operations or program blocks were embedded in the cRIO controllers for processing the waveforms measured in the inverters in real-time, with the help of an integrated FPGA. In parallel, a LabVIEW project is also running on the server to control the information exchange with the cRIO controllers, the WSN, and the Cumulus software.

As mentioned above, a programmable cRIO controller was installed in each of the pilot installation’s four selected inverters, recording current and voltage measurements. The acquisition of data for the PQ analysis was performed through virtual instruments (VI) developed with the NI LabVIEW graphical programming tool and operating in real-time on the cRIO chassis.

According to IEC standard 61000-4-30 [47] for PQ, the basic measurements for detecting events is the RMS value of the signal at each measurement channel for a refreshed half-cycle. It also establishes that the basic time interval for measurements of the amplitudes parameter (supply voltage, harmonics, interharmonics, and unbalance) should be 10 cycles for a network of 50 Hz or 12 cycles for a network of 60 Hz. LabVIEW includes a specific library for analyzing PQ based on the standard [47], which was used in this work. Communication with the FPGA was undertaken via a critical priority loop included in the cRIO, and the PQ measurements were processed in parallel to the data acquisition loop.

Some configuration versions have been developed to verify the most efficient design of the *PV-on time* system. For instance, to exchange information in real-time, several tests were performed with different methodologies, such as local variables, shared variables, and real-time buffers, the last reflecting the more deterministic results. Regarding Ethernet communication, it is optimal to send all the variables in a single packet instead of using multiple datasets. Concerning the real-time processing, the VIs are faster when processing data and sending them to a remote computer for further viewing than if the data are visualized in the same VI.

Finally, these processed PQ data of the cRIO controllers were collected through the Ethernet connection every 200 ms, so it can be said to be real-time. Likewise, the WSN measures were requested every 1 s to the gateway and the weather parameters were read from the Cumulus software register, which is updated every 10 s. All these variables were subsequently employed in non-time-critical calculations.

The architecture of the system allows all registered data to be unified into a single point for processing and for transmission to an endpoint for monitoring and supervision [62]. In the developed system, the acquired data were transmitted [63] to a host computer where a LabVIEW application collects, processes, and analyzes all the measured data. The data were processed in order to obtain useful information both about performances and energy losses in each of the PV system components ([23], pp. 157–158), according to UNE-EN standard 61724:1998 [42], as well as information on the quality of the electrical signal injected into the grid [47], which is indicated in Table 1.

Moreover, in the LabVIEW application, a local monitoring system has been developed. The local monitoring system is responsible for controlling the equipment operation, displaying the status of all the data acquisition devices, and accessing of all the registered, processed, and/or stored information on time.

#### 2.6.2. On Time Monitoring System

Once processed, both records of the measures and the values of the calculated variables are stored in a database that works as a source of information for the remote monitoring application. The Enterprise Information System (EIS) Tier was implemented by a relational database system, specifically a MariaDB server [64], which is responsible for storing both the measured variables and the processed information. The EIS Tier is physically located in the same server as the LabVIEW application, and it is sited to the internal network so the information can only be stored by the processing system and accessed through the business logic. In this way, a high level of security was achieved. The connection between the LabVIEW system and the database engine was performed using an Open Database Connectivity (ODBC) Driver. Therefore, the information could be directly recorded from the Labview application by implementing the different SQL queries.

On the top of the database system, a Java EE application was developed using the open source GlassFish server [65]. Four different components were used in this application. In the bottom layer, the database system was interfaced by means of a native Java database connector (JDBC) which allows the interconnection with the DB engine and the performance of all the queries. The next layer made use of the Java persistence API (JPA) [66] that fetches the database tuples in Java native Objects, so the query and filter operations can be directly programmed in Java. These operations were programmed in the following layer or business logic with the Enterprise Java Beans (EJB) technology, which implements containers managed classes specially designed for a high concurrency and availability.

Finally, the data were made available through a RESTful service based on JAX-RS technology [67]. This implementation allows a universal access to the data since they can be transmitted and requested using the HTTP, or in our case the HTTPS protocol, in order to encrypt the communication, and in the JSON or XML format. In addition, to restrict the access to only authorized users, a password authentication layer was implemented. The RESTful service provides a flexible access to the data and it can be integrated with desktop, smartphones, or tablet applications. In these particular cases, an even more general approach was taken and the back-end RESTful system was interfaced with a web front-end based on the AngularJS google framework [68].

In this way, the monitored data can be supervised from everywhere and in real-time by the granted users, providing they have a device with a web browser. Moreover, the historical registers can be also queried, and the results data represented or exported into CSV files.

## 3. Results

In this section, some results of the developed monitoring system working on the Utility-Scale Power Plant selected are presented.

### 3.1. Real-Time Acquisition in the cRIO Systems

**Figure 16:**
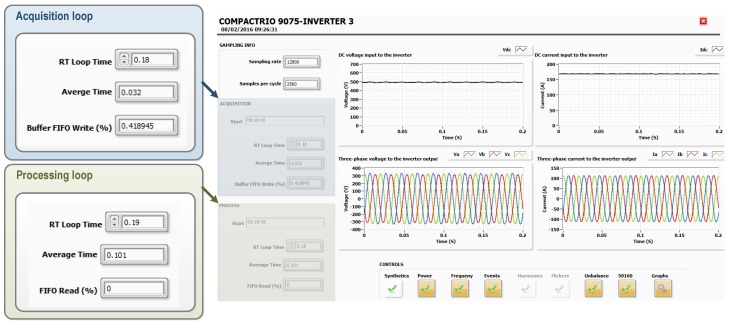
Monitoring PV inverter in real-time.

For the inverter monitoring, a VI is executed in real-time into the cRIO associated. This VI is configured in such a way that the acquisition loop of voltages and currents and the loop processing PQ measures are executed in parallel. Figure 16 shows the VI’s front panel. On the left side, the status of execution of both loops is shown. On the right side, DC input and AC output inverter signals are shown.

To work in real-time and to maintain the determinism, acquisition and processing loops are configured as real-time loops. The acquisition loop is configured to run every 180 ms with the top priority and the processing loop is configured to run every 190 ms with lower priority. The average execution times for both processes, shown in Figure 16, ensure the continuity of the acquisition process. That is, the cRIO system acquires electrical measures from the inverter and processes PQ measures in real-time without losses. For the selected cRIO, model cRIO9075, the processing time for the PQ measurements included in Table 1 is about 100 ms. That is not a closed architecture, so the monitored parameters can be modified and the cRIOs chassis can be replaced for others with better features.

### 3.2. Synchronization and Communication Procedures of Distributed Measurements

In this section, experimental results of the transfer time between two devices using the IEC 61850 standard are shown analyzing the processing times of the senders and receivers under a mode based on the Round-Trip test. A DUT A as a sender and DUT B as a receiver is needed. In this case, the DUT A marks the start transmission time just before issuing the message and the DUT B receives a GOOSE or MMS message to which it was subscribed and processes and marks the exact instant of reception. The time spent since the start of transmission A and the end of reception B represents the total transmission time, including processing A and processing B. This time resembles much more the transmission time defined by the IEC 61850 Part 5 and shown in Figure 15a as $t_{t}$. So, the time allotted to the communication channel is not considered and therefore only takes into account the processing time of the sender, and receiving and processing time of the receiver.

The research was based on the use of data with some complexity, such as including information for periodically transmitting “active power” and one for eventual transmission detecting a “voltage event”. For the analysis of the processing times of the “active power”, a DO TotWh was used and LN of type MMTR (3 Phase Metering) was transmitted. For the analysis of the processing times of the “voltage event”, a DO VarStr (voltage variation Start event in progress) was used, which indicates a current voltage variation, and LN of type QVVR (Voltage variation) was transmitted.

The Table 2 lists the average times obtained from 1000 consecutive measurements. To accurately perform measurements, both teams run on two real-time loops running with an exact frequency of 1 s. Both loops are kept synchronized with an absolute time reference. In Table 2, the level of synchronization of each of the cRIO models is included. The measure is established with respect to a computer acting as master PTP and including a GPS reference.

The DUT is organized into two categories. Classic models that use as RTOS the VxWork, which are models 9024, 9075, and 9074, and the new generation Linux RT, which are models 9030, 9066, and 9033. According to Figure 15a, the total transfer time $t_{t}$ is the sum of the processing time of the message in the DUT A ($t_{a}$), the time that the communications network needed to transmit data ($t_{b}$), and processing time in the DUT B ($t_{c}$). Considering $t_{b}$ null, the communication of an MMS message by a LN MMTR from the cRIO9024 to the cRIO9075 gets a transfer time $t_{t}$ = $t_{a}$ + $t_{c}$ = 1.123 ms + 0.634 ms = 1.757 ms.

Analyzing the times associated with the transmission and reception of data in Table 2, it can be seen that the models based on Linux RT have a shorter response time and greater accuracy in clock synchronization.

Moreover, the management of GOOSE messages developed for real-time transmission can only be generated by devices with Linux RT architecture. The models with real-time operating system VxWork can only manage MMS messages, which are less deterministic but compatible with nodes defined with IEC 61850.

In conclusion, the response time is substantially improved with higher computational capacity. This type of study allows cRIO to choose the appropriate model for each type of application. According to the results obtained, for detection of critical events, it is convenient to select the equipment with higher performance, such as the cRIO 9030 or 9066.

### 3.3. Data Processing System

**Figure 17:**
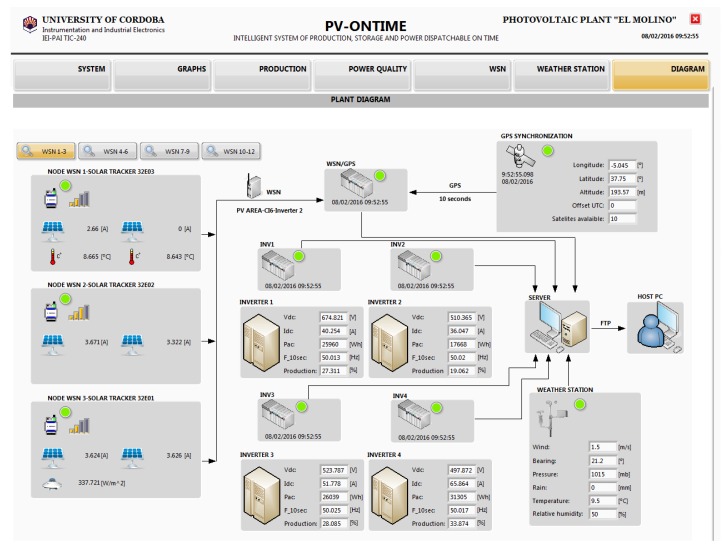
Monitoring application for supervising the *PV-on time* system.

The *PV-on time* system acts as a supervisory control and data acquisition (SCADA) application for the real-time monitoring of the status of data acquisition equipment and the collected measurements. An example of this application is shown in Figure 17.

The top bar shows the menu options which enable the detail panel of the devices or measures groups: “System”, “Graphs”, “Production”, “Power quality”, “WSN”, “Weather station”, and “Diagram”. The figure illustrates the *PV-on time* system where the option “Diagram” is selected. In this screen, a schematic representation of equipment in the monitoring system *PV-on time* is shown.

On the left side, the measures of the WSN nodes installed on the PV plant are shown. There is a menu dedicated to this equipment which enables selection of the WSN nodes in groups of three. The processing software logs link quality and measurement data of all WSN nodes.

The central area displays information about the cRIO programmable controllers, such as the time synchronization. In the case of the cRIOs installed in the TC, the energy and production data are also shown. The production is shown in percentage relative to the nominal power of the PV modules associated with the inverter to which is connected. On the right, data from the GPS system and environmental measures from the weather station are shown.

The data processing system is regularly analyzing the status of all devices. In this screen, device status is represented by a visualization icon (led indicator). For this, the following color code is used: green, executing device; yellow, loss synchronization alarm; red, loss communication alarm; gray, disconnected device. This screen is very useful for viewing the synchronization between equipment.

### 3.4. Monitoring on Time

**Figure 18:**
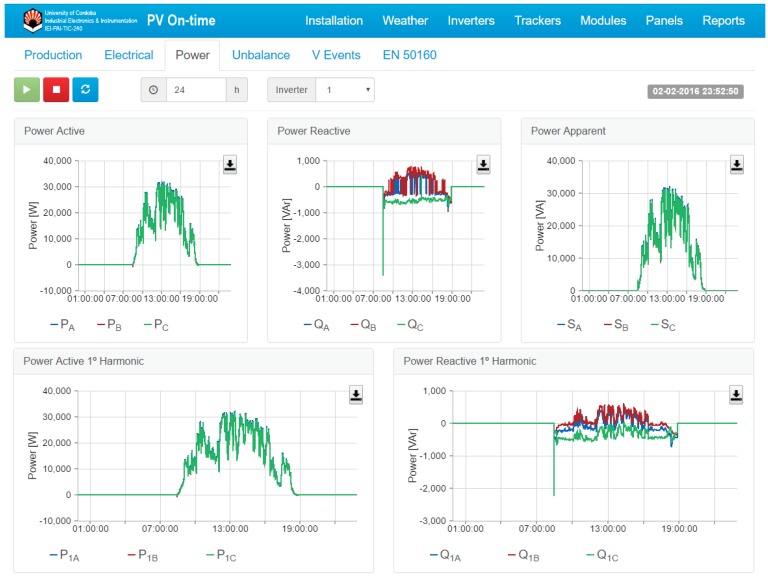
On-line monitoring application for supervising the PV plant in real-time.

All data magnitudes stored in the database can be accessed through The Internet from remote monitoring stations (PCs, mobiles, and tablets). The on-line monitoring application allows fast and intuitive access to all information, presenting data with graphic interfaces and representing instantaneous and averaged measurements interactively. The refresh rate of the web page is set to be 5 s, while the visualization period can be selected in a range from 1 to 24 h. For example, Figure 18 shows the power instantaneously produced during a day by an inverter.

## 4. Conclusions

A new system has been implemented and set up in order to achieve the detailed and comprehensive supervision of all components of a Utility-Scale PV Power Plant connected to the grid. The system, with the incorporation of additional sensors (wired and wireless) and programmable controllers, monitors the production at specific points distributed around the a PV plant’s area. The system can be configured with different sampling times, which renders it highly versatile. The processing of all of the information provides real-time knowledge of the performance of all PV installation components and the presence of any failure in their operation. Due to the higher capacity of the cRIO programmable controllers selected, the PQ of the generated electrical signal can also be analyzed. The monitoring and supervising applications, which were also developed specifically to be integrated into the *PV-on time* system, enables complete real-time monitoring of the plant.

Incorporating the high precision synchronization system, as well as analyzing PQ magnitudes, are the main features that differentiate this system compared to the previously developed ones.

Implemented tests and experimental results showed the correct effectiveness of the embedded system designed for monitoring the PV production, meeting the specifications of several Smart Grid standards. Although the system was configured for a particular Utility-Scale PV Power Plant, it can be easily scalable to different plant topologies and with higher nominal power. An increase in the reliability of the Utility-Scale PV Power Plant components, reducing the operation and maintenance costs could be achieved by developing self-diagnosis algorithms and mechanisms for identifying and classifying deviations and failures in any of the installation’s elements—an easy task with the help of the *PV-on time* system. Likewise, the developed system could be redesigned to be scaled to any number of measurement points, incorporate a production prediction system to compare the results with the actual production of the plant, make a complete system, or obtain low-power and low-cost devices. In addition, when a storage system is incorporated in the plant, the system should also be able to monitor it. Finally, it will also help to automatically obtain information in order to optimize the performance of the PV installation, as well as accommodating production to the grid.

## Figures and Tables

**Table 1:** Real-time processing of power quality measurements in programmable controller.

Measurements	Computed	Intervals
One-cycle fundamental power values	3-phase	1 cycle
Power values	3-phase	10 cycles
Energy values	3-phase	10 cycles
Aggregated frequency 10 s values	1-phase	10 s
Voltage RMS values	3-phase	10 cycles, 3 s, 10 s, 10 m
Current RMS values	3-phase	10 cycles, 3 s, 10 s, 10 m
Voltage underdeviation	3-phase	10 cycles, 3 s, 10 s, 10 m
Voltage overdeviation	3-phase	10 cycles, 3 s, 10 s, 10 m
Voltage events	3-phase	1 cycle
Rapid voltage changes events	3-phase	1/2 cycle
Voltage total harmonic distortion (THD)	3-phase	10 cycles, 3 s, 10 s, 10 m
Voltage unbalance factor	1 value for 3-phase	10 cycles
Fundamental voltage unbalance factor	1 value for 3-phase	10 cycles, 3 s, 10 s, 10 m
Fundamental voltage symmetrical components	1 value for 3-phase	10 cycles
Current unbalance factor	1 value for 3-phase	10 cycles
Fundamental current unbalance factor	1 value for 3-phase	10 cycles
Harmonic voltage RMS values	3-phase	10 m

**Table 2:** Results for data transfer between devices under test (DUT). RTOS: Real-Time Operating System.

	RTOS Linux			RTOS VxWorks		
Definition of the methodology used in the data transfer						
DUT Function	Publish	Subscribe	Subscribe	Publish	Subscribe	Subscribe
cRIO Model	9030	9066	9033	9024	9075	9074
Message type	GOOSE	GOOSE	GOOSE	MMS	MMS	MMS
Evaluation of the times in the data transfer						
PTP synchronism (*μ*s)	32	36	42	66	70	67
Active power (ms)	0.911	0.372	0.453	1.123	0.634	0.560
Sag event (ms)	0.914	0.455	0.513	1.033	0.552	0.510

## Abbreviations

The following abbreviations are used in this manuscript:

AC	Alternating Current
API	Application Programming Interface
ACSI	Abstract Communication Service Interface
CRIO	CompactRIO
CSV	Comma-Separated Values
DA	Data Attributes
DAC	Digital to Analog Converter
DB	Database
DC	Continuous Current
DER	Distributed Energy Resource
DO	Data Objects
DPVA	PV Array characteristics
DPVM	PV Module ratings
DSO	Distribution System Operator
DUT	Device Under Test
EE	Enterprise Edition
EIS	Enterprise Information System
EJB	Enterprise Java Beans
FTP	File Transfer Protocol
GOOSE	Generic Object Oriented Substation Event
GPS	Global Positioning System
GSE	Generic Substation Event
HTTP	Hypertext Transfer Protocol
HTTPS	Hypertext Transfer Protocol Secure
IEC	International Electrotechnical Commission
INV	Inverter
JAX-RS	Java API for RESTful Web Services
JDBC	Java Database Connector
JPA	Java Persistence API
JSON	JavaScript Object Notation
LABVIEW	Laboratory Virtual Instrument Engineering Workbench
LD	Logical Devices
LN	Logical Nodes
MMDC	DC Measurement
MMET	Meteorological Information
MMS	Manufacturing Message Specification
MMTR	Metering 3 Phase
MMXU	Measurement
NI	National Instruments
NIST	National Institute of Standards and Technology
NTP	Network Time Protocol
O&M	Operating and Maintenance costs
ODBC	Open Database Connectivity
PQ	Power Quality
RMS	Root Mean Square
PTP	Precision Time Protocol
PV	Photovoltaic
QFVR	Frequency Variation
QITR	Current Transient
QVTR	Voltage Transient
QVVR	Voltage Variation
RESTFUL	Representational State Transfer
RSYN	Synchronism-check
RTD	Resistance Temperature Detector
RTOS	Real-Time Operating System
SCADA	Supervisory Control And Data Acquisition
SNTP	Simple Network Time Protocol
SPQA	Smart Power Quality Analyzer
SQL	Structured Query Language
ST	Solar Tracker
SV	Sample Value
TC	Transformer Center
THD	Total Harmonic Distortion
TS	Test Set
UTC	Universal Time Coordinate
VARSTR	Voltage variation Start event in progress
VI	Virtual Instrument
WSN	Wireless Sensor Network
XML	Extensible Markup Language
ZINV	Inverter for converting direct current to alternating current (DC→ AC)
